# Integration of gene-based markers in a pearl millet genetic map for identification of candidate genes underlying drought tolerance quantitative trait loci

**DOI:** 10.1186/1471-2229-12-9

**Published:** 2012-01-17

**Authors:** Deepmala Sehgal, Vengaldas Rajaram, Ian Peter Armstead, Vincent Vadez, Yash Pal Yadav, Charles Thomas Hash, Rattan Singh Yadav

**Affiliations:** 1 Institute of Biological, Environmental and Rural Sciences (IBERS), Aberystwyth University, Gogerddan, Aberystwyth, Ceredigion SY23 3 EB, UK; 2 International Crops Research Institute for the Semi-Arid Tropics (ICRISAT), ICRISAT-Patencheru, Hyderabad 502 324, Andhra Pradesh, India; 3 Chaudhary Charan Singh Haryana Agricultural University (CCSHAU), Bawal 123 501, Haryana, India

**Keywords:** CISP, Candidate genes, Drought tolerance QTLs, EST-SSR, Pearl millet, SNP

## Abstract

**Background:**

Identification of genes underlying drought tolerance (DT) quantitative trait loci (QTLs) will facilitate understanding of molecular mechanisms of drought tolerance, and also will accelerate genetic improvement of pearl millet through marker-assisted selection. We report a map based on genes with assigned functional roles in plant adaptation to drought and other abiotic stresses and demonstrate its use in identifying candidate genes underlying a major DT-QTL.

**Results:**

Seventy five single nucleotide polymorphism (SNP) and conserved intron spanning primer (CISP) markers were developed from available expressed sequence tags (ESTs) using four genotypes, H 77/833-2, PRLT 2/89-33, ICMR 01029 and ICMR 01004, representing parents of two mapping populations. A total of 228 SNPs were obtained from 30.5 kb sequenced region resulting in a SNP frequency of 1/134 bp. The positions of major pearl millet linkage group (LG) 2 DT-QTLs (reported from crosses H 77/833-2 × PRLT 2/89-33 and 841B × 863B) were added to the present consensus function map which identified 18 genes, coding for PSI reaction center subunit III, *PHYC*, actin, alanine glyoxylate aminotransferase, uridylate kinase, acyl-CoA oxidase, dipeptidyl peptidase IV, *MADS*-box, serine/threonine protein kinase, ubiquitin conjugating enzyme, zinc finger C- × 8-C × 5-C × 3-H type, *Hd*3, acetyl CoA carboxylase, chlorophyll a/b binding protein, photolyase, protein phosphatase1 regulatory subunit SDS22 and two hypothetical proteins, co-mapping in this DT-QTL interval. Many of these candidate genes were found to have significant association with QTLs of grain yield, flowering time and leaf rolling under drought stress conditions.

**Conclusions:**

We have exploited available pearl millet EST sequences to generate a mapped resource of seventy five new gene-based markers for pearl millet and demonstrated its use in identifying candidate genes underlying a major DT-QTL in this species. The reported gene-based markers represent an important resource for identification of candidate genes for other mapped abiotic stress QTLs in pearl millet. They also provide a resource for initiating association studies using candidate genes and also for comparing the structure and function of distantly related plant genomes such as other Poaceae members.

## Background

Pearl millet [*Pennisetum glaucum *(L.) R. Br.] (2n = 2 × = 14) is the sixth most important global cereal crop (after rice, wheat, maize, barley and sorghum) grown as a rainfed grain and fodder crop in the hottest, driest regions of sub-Saharan Africa and the Indian subcontinent. It produces nutritious grain and is a major human food for people living in the semi-arid, low input, dryland agriculture regions of Africa and South Asia.

Molecular markers-based genetic maps are necessary for applied genetics and breeding programmes of pearl millet. Compared to other cereals such as rice, sorghum, maize, wheat, and barley, there has been relatively little research on the development and application of molecular-markers based genetic maps in pearl millet. Hitherto, genetic maps in pearl millet have been based on markers such as Restriction Fragment Length Polymorphism (RFLP) and Amplified Fragment Length Polymorphism (AFLP) [1-5] with the Simple Sequence Repeat (SSR) and Diversity Array Technolgy (DArT)-based maps [6-8] now being in ascendancy. These maps have proven useful not only in the identification of QTLs and breeding for drought tolerance [4,5,9], disease resistance [10-14] and stover quality [15] but have also improved our understanding of complex relationships between the pearl millet genome and those of other graminaceous species [3]. Despite the availability of a few moderate-density genetic maps and a bacterial artificial chromosome library [16], progress towards identification of genes underlying traits of interest in pearl millet has been hampered by the laborious nature of map-based cloning. To link important agronomical and physiological traits to functional sequence variations and to find candidate genes underlying traits of agricultural interest, there is a need of developing and mapping gene-based markers which currently are in paucity in pearl millet. Pearl millet would benefit greatly from a systematic effort to map functionally important genes to facilitate search for associations between candidate genes and QTLs underlying agriculturally important traits. Molecular variation based on functionally defined genes underlying specific biochemical or physiological functions will provide the next generation of molecular markers for pearl millet. The advantage of such markers, often described as 'candidate gene-based', is their close association with loci controlling variation for the trait in question, allowing the development of 'perfect markers' [17] that can be used for linkage disequilibrium (LD) based mapping studies [18,19] and the direct selection of genotypes with superior allele content [20].

EST resources have proven to be excellent resources for gene discovery, molecular marker development, analysis of gene expression, and identification of candidate genes for phenotypes of interest in a number of species [21-23]. The EST approach is particularly useful for taxa whose genome sequence is presently unavailable or otherwise have limited sequence information. Recently, ESTs have been used for identifying SNPs in many plant species such as rice, maize, barley, soybean, sugarcane, sugar beet and melon [24-30]. The abundance, ubiquity and interspersed nature of SNPs together with the potential of automatic high-throughput analysis make them ideal candidates as molecular markers for construction of high density genetic maps [30], association analysis of candidate genes with important agronomic traits [19,31], fine mapping of QTLs [32], genetic diversity assessment [33,34] and marker-assisted plant breeding [21,35]. In addition, SNPs located in known genes provide a fast alternative to analyse the fate of agronomically important alleles in breeding populations, thus providing functional markers.

In the present study, we have exploited available pearl millet EST sequences to generate a mapped resource of 75 new gene-based markers for pearl millet. Both positional and candidate gene approaches were combined to generate the present gene-based map. The resulting map was used as a template to overlay a major validated DT-QTL [4,5,9] to identify the underlying candidate genes. The presented approach demonstrates how integration of different genomic resources, such as ESTs/genes with traditional genetic and phenotypic data can improve our understanding of complex traits and gene function. Such a molecular map, based on genes with assigned functional roles in plant adaptation to drought and other abiotic stresses, may also be useful for comparing the structure and function of distantly related plant genomes such as other Poaceae members.

## Results

### Length and single nucleotide polymorphism in mapping population parental pairs

A set of 350 gene-specific primers was used to amplify the DNA of four pearl millet inbred lines, H 77/833-2, PRLT 2/89-33, ICMR 01029 and ICMR 01004. One-hundred and ninety (54.3%) of these primer pairs showed strong single reproducible bands on 1% agarose gels. The products of these 190 primers were run on 6% polyacrylamide gel to screen for length polymorphisms. Eighteen and ten primers were polymorphic between H 77/833-2 and PRLT 2/89-33, and between ICMR 01029 and ICMR 01004, respectively. The remaining amplicons which did not detect any length polymorphism in parents but showed single monomorphic band on polyacrylamide gels were sequenced in the four parental genotypes for SNP discovery.

In all, length and (or) sequence polymorphism was detected for 75 markers. The majority of successful assays (84%) detected sequence polymorphisms, while only 16% exhibited length polymorphisms. In total, 30,480 bp of non-redundant sequence data was scanned leading to identification of 228 SNPs with an overall frequency of one SNP per 134 bp (Table 1). The number of SNPs detected between PRLT 2/89-33 and H 77/833-2 (202) was much higher than between parental pair ICMR01029 and ICMR01004 (110) with an SNP frequency of 1/150 and 1/277 bp, respectively. In the total set of SNPs, transitions accounted for 141 (61.8%) and transversions for 87 (38.1%), respectively. Of the 63 gene fragments in which SNPs were discovered, 23 had a single SNP and multiple SNP loci were detected in the remainder (Table 1). The size of indels in CISP markers ranged from 1 to 61 bp (Table 2).

**Table 1:** Marker name, gene homology, total number of SNPs, and number of transitions and transversions obtained in the sequenced region of the gene

SNP marker *	Gene homology	Sequenced region (bp)	Number of SNPs	Number of transitions	Number of transversions
*Xibmsp01*	Ribosomal protein S17 putative	160	1	0	1
*Xibmsp02*	Coproporphyrinogen III oxidase	350	2	1	1
*Xibmsp03*	CorA-like Mg2^+ ^transporter protein	350	3	2	1
*Xibmsp04*	Hypothetical protein	425	5	5	0
*Xibmsp05*	Elongation factor TS	625	6	4	2
*Xibmsp06*	HCO3 transporter family	320	2	1	1
*Xibmsp07*	Serine carboxypeptidase III precursor	710	1	1	0
*Xibmsp08*	Serine carboxypeptidase	200	2	0	2
*Xibmsp09*	Uridylate kinase	680	6	4	2
*Xibmsp10*	Phosphatidylinositol 3-kinase	400	4	3	1
*Xibmsp11*	Acetyl CoA carboxylase	600	1	1	0
*Xibmsp12*	Acyl CoA oxidase	740	2	2	0
*Xibmsp13*	Potassium transporter	780	1	1	0
*Xibmsp14*	Serine-threonine protein kinase	690	1	1	0
*Xibmsp15*	Zinc finger C- × 8-C × 5-C × 3-H type	620	2	2	0
*Xibmsp16*	Pitrilysin	520	3	2	1
*Xibmsp17*	MAP kinase	500	1	0	1
*Xibmsp18*	CBL interacting protein kinase	580	4	2	2
*Xibmsp19*	2-oxoglutarate dehydrogenase E1 component	530	2	1	1
*Xibmsp20*	Succinyl-CoA ligase alpha subunit	590	4	3	1
*Xibmsp21*	Hypothetical protein	300	3	1	2
*Xibmsp22*	*LHY *	570	14	6	8
*Xibmsp23*	Hypothetical protein	200	2	0	2
*Xibmsp24*	Ubiquitin conjugating enzyme	460	2	2	0
*Xibmsp25*	Proteasome a-type and b-type	500	1	1	0
*Xibmsp26*	Catalase	270	4	4	0
*Xibmsp27*	Alanine glyoxylate aminotransferase	880	23	9	14
*Xibmsp28*	Glutaredoxin	750	4	3	1
*Xibmsp29*	Delta-1-pyrroline-5-carboxylate synthetase	680	1	1	0
*Xibmsp30*	*FlO*	330	1	1	0
*Xibmsp31*	*HD3*	370	1	1	0
*Xibmsp32*	Alcohol dehydrogenase 1	370	1	0	1
*Xibmsp33*	ABA response protein	470	3	2	1
*Xibmsp34*	*MADS*-box	330	1	1	0
*Xibmsp35*	*MYC*	370	1	1	0
*Xibmsp36*	Opaque 2	370	1	1	0
*Xibmsp37*	*LEA*	510	6	4	2
*Xibmsp38*	Vacuolar H^+ ^ATPase subunit c	900	4	2	2
*Xibmsp39*	RAB	470	1	1	0
*Xibmsp40*	Anion channel protein	280	4	3	1
*Xibmsp41*	Hydroxyproline rich-glycoprotein	370	1	1	0
*Xibmsp42*	Expressed protein	500	3	2	1
*Xibmsp43*	Actin depolymerising factor	650	8	7	1
*Xibmsp44*	Photolyase	750	3	2	1
*Xibmsp45*	Expressed protein	200	4	4	0
*Xibmsp46*	Plectin/s10 domain	400	12	5	7
*Xibmsp47*	Hypothetical protein	650	8	5	3
*Xibmsp48*	Thioredoxin peroxidase	830	2	1	1
*Xibmsp49*	Atftsh2/8	540	4	2	2
*Xibmsp50*	Fatty acid desaturase	320	1	1	0
*Xibmsp51*	Hypothetical protein	320	5	4	1
*Xibmsp52*	Expressed protein	310	13	6	7
*Xibmsp53*	PSI reaction center subunit III	180	1	1	0
*Xibmsp54*	Eucaryotic initiation factor 4A	750	1	1	0
*Xibmsp55*	*PHYC*	350	3	1	2
*Xibmsp56*	Elongation factor	320	1	1	0
*Xibmsp57*	Zn finger *WRKY*	360	1	1	0
*Xibmsp58*	Fe-S precursor protein	440	1	1	0
*Xibmsp59*	Ycf68	420	1	0	1
*Xibmsp60*	Dipeptidyl peptidase IV	750	3	3	0
*Xibmsp61*	Peroxidase	380	6	4	2
*Xibmsp62*	Actin	530	9	5	4
*Xibmsp63*	AMP deaminase	360	6	3	3

*The sequences of forward and reverse primers are provided in the supplementary Additional file 1: Table S1

**Table 2:** Marker name, gene homology, and size of Indel polymorphism for the CISP markers in parents H 77/89-33 and PRLT 2/89-33

CISP marker*	Gene homology	Size of Indel
*Xibmcp01*	Heat Shock protein	9 bp
*Xibmcp02*	Ribosomal protein L24	12 bp
*Xibmcp03*	Transmembrane amino acid transporter	12 bp
*Xibmcp04*	Transaldolase	61 bp
*Xibmcp05*	C2 domain	6 bp
*Xibmcp06*	Adenosyl homocysteinase	4 bp
*Xibmcp07*	Phosphate translocator	5 bp
*Xibmcp08*	Phosphoglycerate kinase	2 bp
*Xibmcp09*	Chlorophyll A/B binding protein	1 bp
*Xibmcp10*	Delta-1-pyrroline-5-carboxylate synthetase	1 bp
*Xibmcp11*	Protein phosphatase 1 regulatory subunit SDS22	3 bp
*Xibmcp12*	Expressed protein	2 bp

*The sequences of forward and reverse primers are provided in supplementary Additional file 2: Table S2

### Linkage mapping

**Figure 1:**
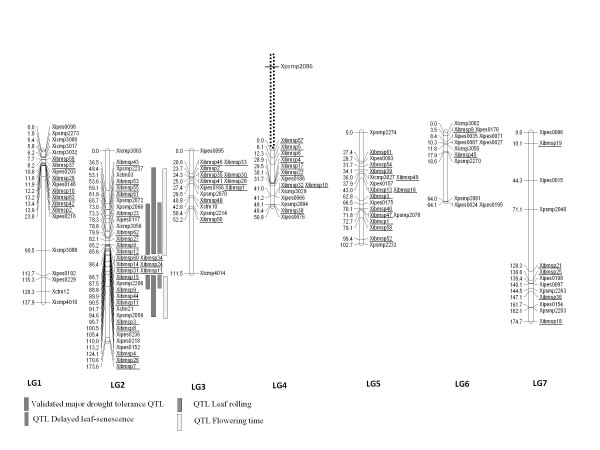
**Pearl millet consensus function map based on gene-based SNPs, CISPs and EST-SSRs**. Distances are given in Haldane cM on the left side of each linkage bar. Candidate genes integrated as SNP and CISP markers are shown as underlined. Major QTLs of drought tolerance added onto the consensus map from Yadav et al. [4,5] and Bidinger et al. [9] are indicated as hatched boxes on the right side of LG2. Six SNP markers, *Xibmsp60, Xibmsp34, Xibmsp14, Xibmsp24, Xibmsp11 *and *Xibmsp31*, showed complete linkage on LG2. Similarly, on LG3 three pairs of SNP loci *Xibmsp46 *and *Xibmsp33, Xibmsp35 *and *Xibmsp30*, and *Xibmsp41 *and *Xibmsp28 *showed complete linkage. On LG5, the SNP markers *Xibmsp13 *and *Xibmsp16 *were completely linked. Complete linkage between gene-based SNPs and framework markers was observed on LG3 (*Xibmsp1 *and *Xipes0166*), LG4 (*Xibmsp32, Xibmsp10 *and *Xicmp3029*), LG5 (*Xicmp3027 *and *Xibmsp49*, and *Xpsmp2078 *and *Xibmsp47*) and LG6 (*Xibmsp8 *and *Xipes0176*). In agreement with the previous studies [6], genomic SSR marker *Xpsmp2086 *showed weak linkage and/or aberrant behaviour i.e. tripled the length of LG4 when incorporated based on its expected position (shown as dashed line in Figure 1). Four CISP markers, *Xibmcp5, Xibmcp6, Xibmcp7 *and *Xibmcp12*, and two SNP markers *Xibmsp20 *and *Xibmsp56 *remained ungrouped.

All the polymorphic markers identified in this study segregated in a co-dominant manner. A total of 133 markers (including 64 framework SSR markers) were assigned to seven linkage groups (Figure 1), designated as LG1-LG7 corresponding to the reference map. The gene-based SNP and CISP markers were distributed on all seven linkage groups. The map of LG2, the main target of this study, where a major DT-QTL of pearl millet resides, was saturated with 24 new gene-based markers of which 20 were SNPs and 4 were CISPs. Most importantly, 18 new gene-based markers were mapped within the support interval of the validated major DT-QTL region (between markers *Xpsmp2237-Xpsmp2059*; Figure 1) on LG2 which originally had only five EST-SSR markers loci mapping across this interval [36]. The least (2) number of genes were mapped on LG6. Individually, LG1, LG2, LG3, LG4, LG5, LG6 and LG7 were mapped with 7, 24, 10, 9, 12, 2 and 5 new gene-based markers, respectively. The total map distance of the combined SSR, SNP and CISP marker map was 815.3 cM, with lengths of individual linkage groups ranging from 50.8 cM (LG4) to 174.7 (LG7).

### Allele frequencies in the H 77/833-2 × PRLT 2/89-33 RIL population

Interestingly, distorted segregation ratios were evident on almost all linkage groups for both framework SSR loci as well as SNP and CISP markers loci. Within each segregation distortion region (SDR), distortion was unidirectional, favouring alleles exclusively from one parent. For instance, on LG1 there is a major region of segregation distortion between *Xibmsp42 *and *Xipes0098*. PRLT 2/89-33 alleles are overrepresented in this distorted region as compared to region between *Xctm12 *and *Xicmp3088 *where H 77/833-2 alleles are preferred. Significant regions of distortions with preference for PRLT 2/89-33 alleles were also noted on LG3 (between *Xibmsp50 *and *Xipes 0095*), LG4 (between *Xipes018*6 and *Xipes0076*), LG5 (between *Xpsmp2274 *and *Xibmsp 40*) and LG6 (between *Xicmp3002 *and *Xpsmp2270*). On LG7, a modest but consistent segregation distortion was observed between *Xipes0198 *and *Xpsmp2203 *and transmission frequency was again higher for alleles of male parent PRLT 2/89-33. On LG2, on the other hand, the H 77/833-2 alleles were more prominent between *Xibmsp53 *and *Xibmcp3*.

### Validation of gene-based markers with the major DT-QTL on LG 2 using fine mapping population

Large number of markers (*Xibmsp27, Xibmsp9, Xibmsp12, Xibmsp60, Xibmsp34, Xibmsp14, Xibmsp24, Xibmsp31, Xibmsp11, Xibmsp15, Xibmcp9, Xibmsp44 *and *Xibmcp11*) mapping to LG 2 showed significant association with yield and yield components, flowering time, delayed leaf senescence and leaf roll under drought stress in the fine mapping population. For illustrating that the markers developed in this study co-map with the DT phenotype, only grain yield, flowering time and leaf rolling data is presented (Table 3). A detailed dissection of other yield and physiological parameters of the DT-QTL using these markers is currently underway and will be reported separately (manuscript under preparation).

**Table 3:** Gene-based markers that segregated with grain yield, flowering time and leaf rolling scores in the fine mapping population (ICMR 01029 × ICMR 01004)

Grain yield	Flowering time	Leaf rolling	Gene name	Synonymous/non-synonymous
Xibmsp27*	Xibmsp27**		Alanine glyoxylate aminotransferase	Synonymous
Xibmsp9*	Xibmsp9***		Uridylate kinase	Synonymous
Xibmsp12**	Xibmsp12***		Acyl CoA oxidase	Non-synonymous
Xibmsp60***		Xibmsp60*	Dipeptidyl peptidase IV	Synonymous
Xibmsp34***	Xibmsp34***		*MADS*-box	Non-synonymous
Xibmsp14***	Xibmsp14***	Xibmsp14*	Serine-threonine protein kinase	Synonymous
Xibmsp24***	Xibmsp24***	Xibmsp24*	Ubiquitin conjugating enzyme	Synonymous
Xibmsp31***	Xibmsp31***	Xibmsp31*	*HD3*	Synonymous
Xibmsp11***		Xibmsp11*	Acetyl CoA carboxylase	Non-synonymous
Xibmsp15***	Xibmsp15***	Xibmsp15*	Zinc finger C- × 8-C × 5-C × 3-H type	Non-synonymous
Xibmcp9**			Chlorophyll A/B binding protein	Non-synonymous
Xibmsp44***		Xibmsp44*	Photolyase	Synonymous
Xibmcp11***			Protein phosphatase 1 regulatory subunit SDS22	Non-synonymous

*P ≤ 0.05

**P ≤ 0.01

***P ≤ 0.001

## Discussion

Due to the multiplicity of genes and their partial effects on phenotypic variation, the candidate gene approach is suggested to be more suitable for QTL characterization than genome wide scanning or positional cloning [37,38]. Molecular-linkage maps based on functional gene markers (molecular-function maps) are a prerequisite for a candidate-gene approach to identify genes responsible for quantitative traits at the molecular level. Therefore, plant function maps have recently been generated in many species [28-30] and candidate genes have been identified for various biotic stress resistance and abiotic stress tolerance QTLs using this approach [39-43]. Considering this, and the fact that hitherto little information on gene-based SNPs is available in pearl millet [44], the present study was undertaken to develop a resource of mapped gene-based SNPs for pearl millet and to identify putative candidate genes underlying a major validated drought tolerance QTL.

We placed 69 gene-based SNPs and CISPs onto existing SSR-based skeleton map of pearl millet based on the cross H 77/833-2 × PRLT 2/89-33. Although the number of markers mapped earlier on this cross is relatively large, a high percentage of the markers are anonymous sequences and/or exhibit dominant patterns of inheritance [1,6,8]. Recently, attempts have been made to enrich the existing pearl millet maps with co-dominant genomic and EST-derived SSR markers [6,7]. The limitation of genomic SSRs is their low cross-species transferability due to either disappearance of the repeat region or degeneration of the primer binding sites. Although cross-species PCR amplification of EST-SSRs is more successful compared with genomic SSRs, their polymorphism rates are, however, very low. The SNP markers, as developed in this study, provide many benefits over SSRs, including their abundance in the genome, frequent occurrence in coding regions of the genes, and their ease of analysis and unambiguous results across various platforms [35,45]. Every SNP in single copy DNA is potentially a useful marker.

A total of 228 SNPs were obtained in 30.5 kb sequenced region resulting in an SNP frequency of one SNP per 134 bp (Table 1). In maize, an out-crossing species like pearl millet, SNP frequency of one SNP per 61 bp was observed with 18 gene fragments analysed in 38 inbred lines [24]. In another study on maize, SNP frequency of 1 SNP per 73 bp was observed with analysis of 592 unigenes in 12 inbred maize lines [46]. For out-breeding forage grass *Lolium perenne*, a frequency of 1 SNP per 54 bp was observed in the analysis of 100 candidate genes [47]. In rye and sugar beet, the estimated SNP frequency was 1 SNP per 58 bp [48] and 1 SNP per 72 bp [41], respectively. Thus, the polymorphism frequency determined for pearl millet is lower than those of other out-crossing species. Selection of germplasm is one of the major factors that affect SNP frequency. A contrasting variation in SNP frequency was reported in two different sets of germplasm of maize having different number of accessions [24,49]. Higher SNP frequencies have generally been reported in studies involving a large number of diverse accessions [24,26,48,50]. Compared to other studies, the number of genotypes used for SNP discovery was very small in the present study. Moreover, two (ICMR 01029 and ICMR 01004) of the four pearl millet genotypes used in this study are QTL near-isogenic lines generated in the background of H 77/833-2. The other important factor that affects SNP frequency between different plant species is the differences in genomic region(s) assayed e.g., coding regions, promoters, introns or untranslated regions (UTRs) [51]. The sequence set in the present study is not complete with respect to any of these categories; in general, this study targeted both exonic regions and 3' -UTRs. Considering this and the fact that the limited number of EST sequences were analysed, the frequency estimates in this study might not reflect the exact picture for pearl millet.

The level of attrition from marker discovery to genetic map assignment in the present study was observed for only 8 SNPs (13.3%). In *Lolium perenne*, the same approach led to assignment of only 40% of the genes on the genetic map [47]. The addition of gene-based markers extended the genetic map of pearl millet by 125 cM; the total length of the combined SSR, SNP and CISP marker-based map being 815.3 cM. Most importantly, 63 gene-based markers mapped to positions different to framework markers, thus enriching the map with new candidate gene loci (Figure 1). Further, some of these candidate gene loci filled the large gaps present in some linkage groups of the framework map. For instance, the framework map had the largest (54.8 cM) gap on LG4 between markers *Xpsmp2086 *and *Xipes0186*. Six gene-based markers (*Xibmsp57, Xibmsp5, Xibmsp6, Xibmcp4, Xibmsp17*, and *Xibmsp22*) mapped between *Xpsmp2086 *and *Xipes0186 *on LG4 (Figure 1).

Interestingly, the recombinant inbred line (RIL) mapping panel used in the present study revealed high rates of SDRs with both framework SSRs and gene-based SNP and CISP markers. Distortions toward either of the parental allele were observed. Such segregation distortion due to an excess of one of the parental allele has also been reported in essentially all previous studies in pearl millet [5-7,9]. Qi et al. [6], for instance, observed SDRs due to an excess of one of the parental allele on LG 4 in the cross 81B × ICMP 451, on LG 4, LG5 and LG7 in LGD 1-B-10 × ICMP 85410, on LG2 and LG4 in PT 732B × P1449-2, and on LG3 and LG6 in ICMB 841 × 863B. However, segregation distortion in the RILs used in the present study was significantly higher compared to all previous studies in pearl millet where F_2 _populations were used [5-7,9]. Higher segregation distortions in RILs compared to F_2 _or other early generations has been reported in other crops as well [52,53]. For example, in a comparative study carried out to explore segregation distortion of molecular markers in different mapping populations (F_2_, backcross, doubled-haploid and RILs) in rice, consistently more segregation distortion was found in RILs than in doubled-haploid, backcross, or F_2 _populations [52]. It has been suggested that more generations result in a stronger effect of selection and therefore segregation distortion accumulates with additional generations of meiosis [53]. Preferential transmission of parental alleles could be caused by an allele-specific advantage in viability or fertility, and gene-based markers may represent or be linked to alleles selected during the six generations used for development of the RILs.

The LG2, the prime target of this study, was saturated with 24 new gene-based markers (Figure 1). Of these, 20 were SNPs derived from genes coding for actin depolymerising factor, PSI reaction centre subunit III, *PHY C*, actin, alanine glyoxylate aminotransferase, uridylate kinase, acyl CoA oxidase, dipeptidyl peptidase IV, Zn finger C × 8-C × 5-C × 3-H (or CCCH type), serine/threonine protein kinase, a homolog of rice flowering time gene *HD3, MADS*-box, acetyl CoA carboxylase, ubiquitin conjugated enzyme, photolyase, catalase, serine carboxypeptidase III precursor and three hypothetical proteins. The four CISP markers on LG2 represented genes coding for a chlorophyll a/b binding protein, protein phosphatase 1 regulatory subunit SDS22, transmembrane amino acid transporter and a phosphoglycerate kinase. The pearl millet SNP map for LG2 was generally consistent with chromosome-level pearl millet-rice synteny (pearl millet LG2 syntenic to rice chromosomes 2S, 3L, 6S and 10S) previously determined with RFLP markers [3]. For example, three genes retrieved from rice chromosome 2S (serine/threonine-protein kinase, LOC_Os02g57080; serine carboxypeptidase III precursor, LOC _Os02g02320; phosphoglycerate kinase, LOC_Os02g07260) and three others from 6S (acyl CoA oxidase, LOC_Os06g01390; zinc finger C- × 8-C- × 5-C- × 3-H type, LOC_Os06g21390; transmembrane amino acid transporter, LOC_Os06g12320) mapped on LG2 of pearl millet. In addition to LG2, pearl millet-rice synteny observed in the present study was also consistent with previous study [3] for other linkage groups. However, a few loci retrieved from rice mapped to nonsyntenic positions on pearl millet linkage groups. For example, uridylate kinase and acetyl CoA carboxylase from rice chromosomes 1S and 5S, respectively, mapped on LG2 of pearl millet. Similarly, vacuolar ATPase subunit C from rice chromosome 5 L mapped on LG4 of pearl millet. Such observations have been reported in other crops such as barley [28] and sorghum [54] and are not surprising given that the rice genome has undergone large segmental, as well as individual gene duplications, mostly after the divergence of rice and Triticeae ancestors.

Exploiting markers common between the present consensus map and other linkage maps of pearl millet, the position of major DT-QTLs have been added to the present function map to identify potential candidate genes for drought tolerance (Figure 1). Eighteen gene-based markers were localised in the support interval of major DT-QTL region on LG2 (Figure 1). Of these, ten genes have been reported to play important roles in regulation [transcription factors like zinc finger C × 8-C × 5-C × 3-His type (or CCCH type), *MADS*-box], signal transduction (serine/threonine protein kinase, protein phosphatase 1 regulatory subunit SDS22), energy and carbon metabolism (genes for photosynthesis, photorespiration and β-oxidation such as PSI reaction center subunit III, chlorophyll a/b binding protein, alanine glyoxylate aminotransferase, acyl CoA oxidase), purine and pyrimidine nucleotide biosynthesis (uridylate kinase), and lipid biosynthesis (acetyl-CoA carboxylase) under drought and osmotic stresses [55-63]. The presence of transcription factors belonging to Zn finger CCCH type and *MADS*-box gene families in support interval of major DT-QTL region is noteworthy. These transcription factors gene families have been reported to activate cascade of downstream genes that act together in enhancing tolerance to multiple stresses [57,61-65]. Among the different types of Zn finger families, role of C_2_H_2 _type Zn finger gene families in drought stress tolerance has been functionally validated in rice and Arabidopsis [64,65]. However, CCCH types Zn finger proteins are poorly characterized in plants under drought stress. The best characterized CCCH-type zinc finger proteins in plants are OsDOS in rice [66], AtSZF1 and AtSZF2 in Arabidopsis [56] and GhZFP1 in cotton [67] under salt and fungal stresses. The CCCH type Zn finger in pearl millet shows significant homology with RING finger types OsC_3_H_41 _and AtC_3_H_69 _of rice (BlastX; 2e-100) and Arabidopsis (BlastX; 1e-65), respectively, the members of which have been reported to be regulated by various biotic and abiotic stresses including water stress induced by mannitol [68].

The *MADS*-box family, identified initially as floral homeotic genes, is one of the most extensively studied transcription factor gene families in plants [57,69]. The most striking feature of the *MADS*-box gene family is the diverse functions taken up by its members in different aspects of plant growth and development including flowering time control [69]. Different members of MADS family have been reported to be induced under drought stress in rice [57,70], maize [71] and wheat [72]. The MADS box gene in pearl millet shows significant homologies with MIKC type MADS-box genes of *Triticum aestivum *(BlastX; 2e-15), *Zea mays *(*MADS22*, BlastX; 2e-15) and *Brachypodium *(*MADS22*, BlastX; 4e-15). The homologue of MADS22 in rice, *OsMADS22*, has been reported to be up-regulated by more than two-fold in response to dehydration stress [57]. In pearl millet, polymorphism in the *MADS*-box gene *MADS11 *has been reported to be associated with flowering time variation [73]. Studies have shown that a large number of genes involved in flower development are associated with abiotic stress responses [74,75]. The significant association of MADS box gene with flowering time and grain yield QTLs in pearl millet under drought stress (Table 3) suggests this to be another strong candidate gene for DT-QTL in pearl millet.

Similarly, the candidacy of serine/threonine protein kinase and acyl CoA oxidase in the DT-QTL interval is supported by expression evidences of these genes in pearl millet [76]. A ~10 fold increase in expression was obtained for both serine/threonine protein kinase and acyl CoA oxidase in pearl millet seedlings subjected to drought stress [76]. Another important gene mapped in the support interval of major DT-QTL was that coding for acetyl-CoA carboxylase (ACC). In plants, ACC isozymes provide malonyl CoA pools used for *de novo *fatty acid synthesis in plastids and mitochondria, and for fatty acid elongation, flavonoid and stilbene biosynthesis in the cytosol [77]. ACC reaction is the most important regulatory step, controlling metabolite flow in response to stress. From the water-deficit stress tolerance perspective, fatty acids are essential in membrane biogenesis, lipoic acid and cuticular wax synthesis and stress signalling [78].

The candidate genes, identified in the present study, were significantly associated with QTLs of grain yield, flowering time and leaf rolling under drought stress (Table 3) thus confirming their associations with drought tolerance phenotype(s) in pearl millet (Table 3). Such mapping of candidate genes also offer a range of possible links to the other physiological [79] and agronomical [4,5,15] traits including salt tolerance [80] that co-map with the major LG2 DT-QTL region. Similar approach has been used in other crops to find positional candidate genes underlying QTLs [28,40,42,81]. For example, a molecular function map with 85 loci was constructed in potato based on 69 genes involved in carbohydrate metabolism and transport to identify the candidate genes for tuber starch content [81]. Similarly, 16 transcription factor genes were integrated on barley framework map and drought and cold tolerance QTLs were positioned on the consensus map to find positional candidate transcription factors for drought and cold tolerance [42]. In the latter study, emphasis was given to transcription factors and upstream regulators, rather than to structural genes. In the present study, however, we have assembled sequences from both structural and transcription factor genes to gain a more complete picture of the distribution of abiotic stress genes around pearl millet DT-QTL interval and across the genome.

## Conclusions

The present molecular function map of pearl millet represents an important step towards identification of candidate genes for abiotic stress QTLs and for other agriculturally important traits that have been mapped in this species. Further, it provides a means to anchor maps across different pedigrees and the basis for *in silico *comparative genetic mapping. The positions of previously reported major QTLs for drought tolerance on the present map have revealed some interesting positional candidates. Currently, we are studying polymorphisms of these LG2 candidate genes in a pearl millet inbred germplasm association panel which will further validate their associations with drought tolerance related traits across wider germplasm of the species. Our future work also involves functional validation of these candidate genes using approaches such as over-expression, anti-sense suppression and (or) double-stranded RNA interference in knock-out mutants and transgenics.

## Methods

### Plant material and mapping population

Pearl millet inbred lines used for SNP and CISP discovery were: H 77/833-2, PRLT 2/89-33, ICMR 01029 and ICMR 01004. PRLT 2/89-33 (terminal drought tolerant) is known for its better grain-filling ability under terminal drought stress conditions. H 77/833-2 (terminal drought sensitive) is the male parent of a number of thermo-tolerant, extra-early, high-tillering and high-yielding pearl millet hybrids formerly grown in north-western India. ICMR 01029 is a near-isogenic line, introgressed with alleles from PRLT 2/89-33 for a major drought tolerance QTL on LG2 in the background of H 77/833-2 by four generations of marker-assisted backcrossing. ICMR 01004 is another QTL introgression line developed in the background of H 77/833-2 by marker-assisted backcross transfer of downy mildew resistance QTLs from LG1 and 4 [82]. An F_6 _RIL population, derived by single-seed descent from a single F_1 _plant from cross H 77/833-2 × PRLT 2/89-33, was used for mapping of gene-based markers. Eighty eight RILs were employed for population screening and map construction.

A high resolution cross (HRC), developed for fine mapping of the major drought tolerance (DT) QTL by crossing two near isogenic lines (NILs; ICMR 01029 and ICMR 01004), segregating for DT-QTL region on LG 2 [82] was used to confirm the association of the gene-based markers developed in the present study with the DT-QTL. Briefly, ~ 2500 individuals of HRC were genotyped with 6 SSR markers covering the entire DT-QTL region on LG2 and 160 most informative selected recombinants were genotyped with gene-based markers developed in this study for the target DT-QTL region, and phenotyped for their response under drought stress conditions [82].

Genomic DNA of the parents and the RILs was extracted using DNeasy plant DNA kit (Qiagen, Hilden, Germany).

### Primer designing

Our main objective was to generate gene-based markers for fine mapping of the validated major DT-QTL on LG2 originally detected using testcross progeny from cross H 77/833-2 × PRLT 2/89-33 [4]. To saturate LG2 with gene-based markers, we utilized published information of synteny between pearl millet LG2 and rice chromosomes 2S, 3 L, 6S and 10 L [3,82]. The genomic sequences of 100 selected genes within the rice bacterial artificial chromosome (BAC) contigs from each of the four syntenic rice chromosomes (between rice markers C1246 and C630 on 2S, C136 and RZ624 on 3 L, PSR 490 and C235 on 6S and between R2447 and C1361 on 10 L) were retrieved using the TIGR Rice Genome Annotation Project portal http://blast.jcvi.org/euk-blast/index.cgi?project=osa1. Majority of these genes are associated with cellular metabolism, signal transduction and/or transcriptional regulation. Primer pairs were designed manually for genes which showed significant homologies (with an e value of 1e-10) with pearl millet ESTs. After predicting exon-intron boundaries using MACAW version 2.05, primers were designed with an average length of 20 nucleotides, GC content of 50 ± 5%, a melting temperature around 60°C and an expected PCR product of 400-800 covering both exons and introns. Primer quality such as 3' end complementarity, presence of hairpin loops was assessed using an on-line oligonucleotides calculator http://www.basic.nwu.edu/biotools/oligocalc.html. For genes where available pearl millet EST sequence was not helpful for primer design, sorghum and maize ESTs (identified using *all transcripts on *option in the TIGR Rice Genome Annotation Project portal) were used to design appropriate primers.

In addition to saturating LG2 with gene-based markers, we also intended to have a SNP map based on abiotic stress responsive genes that could be candidates underlying biotic or abiotic stress QTLs previously reported in pearl millet [4,5,9-14]. To identify such genes, we used another *in silico *approach wherein we identified 200 pearl millet ESTs from the NCBI http://www.ncbi.nlm.nih.gov/ database that were homologous to drought and other abiotic stress genes reported widely in other model or non-model crops using the BLAST2GO programme http://www.blast2go.org/. For this set of 200 ESTs, primers were designed using Batch Primer 3 software http://probes.pw.usda.gov/batchprimer3/index.html to amplify an average region of 400 bp that covers part of the 3' untranslated region (3'UTR). The 3'UTR regions are expected to have greater sequence polymorphisms and therefore the 3'-most 500 bp were targeted in primer design. Primers were designed to have an average length of 20 nucleotides, melting temperatures of 58°C or 60°C, and theoretical PCR amplicons of 150-600 bp. In addition, a set of 50 published CISP primers [83] were also tested to find SNPs and insertion-deletions (Indels) among the four genotypes (H 77/833-2, PRLT 2/89-33, ICMR 01029 and ICMR 01004).

### PCR amplification

All PCR reactions were performed using 20 ng of genomic DNA in a 20 μl PCR reaction mix containing 1 unit of *Taq *polymerase, 1.5 mM MgCl_2_, 100 μM of each of the four dNTPs, and 5 pmol each of forward and reverse primers. The PCR cycling used for most of the primer pairs included an initial denaturation step at 95°C for 3 min, followed by 10 cycles of touch down at 95°C for 20 s, from 63 to 58°C for 20 s (0.5°C decrease per cycle), and 72°C for 80 s. The touch down was followed by 36 cycles of 95°C for 20 s, 58°C for 20 s, and 72°C for 80 s. In situations where these general conditions did not work, they were modified by extending the number of cycles to 40 (without the initial touch down cycles) and by varying the annealing temperatures from 55 to 60°C.

### Detection of gene polymorphism and genotyping

PCR products were resolved on 6% non-denaturing polyacrylamide gels and visualized by silver staining [84]. Amplification products that did not show length polymorphism between the four parents were selected for sequencing. For sequencing, the PCR product was purified with QIAquick PCR purification kit (Qiagen, UK). Sequencing was performed using the ABI Prism BigDye Terminator Cycle Sequencing kit (Applied Biosystems, CA, USA) with an ABI Prism 377 genetic analyser (Applied Biosystems). PCR products were sequenced in both forward and reverse orientation. Sequences obtained from four genotypes (H 77/833-2, PRLT 2/89-33, ICMR 01029 and ICMR 01004) were aligned using MACAW 2.05 software, and SNPs were identified. Putative SNP positions were visually verified directly on the sequence chromatograms using the Chromas 1.45 programme. Heterozygous loci from parental lines were excluded from SNP identification.

For genotyping the mapping population with CISP markers (size polymorphic between parents on PAGE), an 18-nucleotide M13 tail sequence (5'CACGACGTTGTAAAACGAC3') was added at the 5' terminus of the forward primers to facilitate labelling of PCR products with a fluorophore- labelled M13 primer. The fluorophores used were 6-FAM, NED, VIC, and PET (Applied Biosystems, Foster City, CA, USA). The programme used for M13-tailed PCR reaction included denaturation at 94°C for 10 min followed by 30 cycles of 30 s at 94°C, 45 s at 56°C, 45 s at 72°C, further followed by 8 cycles of 30 s at 94°C, 45 s at 53°C, 45 s at 72°C ending with extension step at 72°C for 10 min. After the PCR products were resolved on an ABI 3730 DNA sequencer (Applied Biosystems, Foster City, CA, USA), the GeneMapper program, version 3.7 (Applied Biosystems, Foster City, CA, USA), was used for scoring alleles.

For SNP genotyping, only one SNP per SNP-containing loci was used for genotyping using KASPar technology (K Biosciences, UK).

### Genetic mapping, comparisons with QTL positions and marker-trait association analysis

Genotyping data for both SNP and CISP markers was generated on 88 RILs of the mapping population. Marker genotyping data was analysed using the *χ*^2 ^test to assess the goodness-of-fit to the expected 1:1 segregation ratio for each marker. Subsequently, genotyping data for all the markers, including those with distorted segregation, were used for linkage analysis using JoinMap software, version 3.0 [85]. A skeleton linkage map of genomic and EST-SSR markers for the (H 77/833-2 × PRLT 2/89-33)-based RIL mapping population [36] was used for identification and orientation of the seven pearl millet linkage groups. Markers were grouped at LOD = 6, and map positions were determined in each linkage group using the Haldane function. All recombination rates up to r = 0.4999 within a linkage group were taken into account to allow the inclusion of more distant loci that are not closely linked, therefore also the LOD threshold for map calculation was set to the minimum of 0.0001. Further selected parameters include a RIPPLE value of 1, a JUMP THRESHOLD of 5, and a TRIPLE THRESHOLD of 5. The order of markers within each group was again determined using MAPMAKER 3.0 at LOD 3.0, and the orders were tested using the ripple command. Maps were then drawn with the program MapChart [86]. The identity and polarity of the seven linkage groups was determined using previously mapped SSR loci [6,7].

Allele frequencies were calculated for each marker locus to evaluate the degree of deviation from the expected 0.5 transmission frequency for each parental allele at each genotyped locus across the RIL population. All markers with segregation data were subjected to this analysis, including the SSR markers of the skeleton map.

Through the relationships with markers common between the present map and published linkage maps of pearl millet, the position of major DT-QTLs on LG2 have been added to the map. Association, if any, of the gene-based markers (developed in this study) to the drought tolerance phenotype was sought using a fine mapping population specifically developed to fine map the drought tolerance QTL region on LG 2 [82]. Marker-trait analysis in the F_2 _fine mapping population was done using ANOVA feature in MINITAB (ver. 14). For each marker the recombinants were grouped into 3 genotype classes based on marker genotype (AA, BB and HH; A being ICMR 01029 allele, B being ICMR 01004 allele, and H being heterozygous). The phenotypic means of these genotypic groups were then compared by one way ANOVA for each of the 26 markers mapping in DT-QTL region.

## Abbreviations

DT: Drought tolerance; QTL: Quantitative trait loci; SNP: Single nucleotide polymorphism; CISP: Conserved intron spanning primer; RFLP: Restriction fragment length polymorphism; AFLP: Amplified fragment length polymorphism; SSR: Simple sequence repeats; DArT: Diversity Array Technology; EST: Expressed sequence tag; LG: Linkage group; SDR: Segregation distortion region; RIL: Recombinant inbred line; HRC: High resolution cross.

## Authors' contributions

DS designed gene-based markers, carried out all the experimental and analysis work, constructed the integrated map and wrote the manuscript; VR developed EST-SSRs; IPA contributed in map construction; CTH provided the framework EST-SSR map and edited the manuscript; VV and YPY read the manuscript critically and contributed in discussion on traits mapping with gene based markers; RSY participated in its design and helped draft the manuscript. All authors have read and approved the manuscript.

## Supplementary Material

Additional file 1 **Table S1**. Forward and Reverse pair of primer sequences used for developing SNP markers from within the genes. Click here for file

Additional file 2 **Table S2**. Forward and Reverse pair of primer sequences used for CISP markers development. Click here for file
