# Supplementary material for: Integration of gene-based markers in a pearl millet genetic map for identification of candidate genes underlying drought tolerance quantitative trait loci
Source: BMC Plant Biol. 2012 Jan 17;12:9. doi: 10.1186/1471-2229-12-9 (PMC3287966; doi:10.1186/1471-2229-12-9)
Supplement: Additional file 1 — Table S1. Forward and Reverse pair of primer sequences used for developing SNP markers from within the genes. [file 1471-2229-12-9-S1.DOCX]

Table S1 Forward and Reverse pair of primer sequences used for developing SNP markers from within the genes

| SNP marker | Gene homology | Forward | Reverse |
| --- | --- | --- | --- |
| *Xibmsp01* | Ribosomal protein S17 putative | GCAGACTGAGAAGGCTTTCC | TGCTCTTCCAGAAGCGGTTG |
| *Xibmsp02* | Coproporphyrinogen III oxidase | GGAGTACAGAGTCCGCACATT | CTTCTCAACTTTGCGACAGGT |
| *Xibmsp03* | CorA-like Mg2^+^ transporter protein | CGCAACAGAATTTTGTCGG | TTACGCTGGTTGTCAAGTTG |
| *Xibmsp04* | Hypothetical protein | AGTGAGTCAAGATCTTCATTTTTCC | AAGGGAATGGCTTGAAGATT |
| *Xibmsp05* | Elongation factor TS | TCTCCTTCTCCTTGCTGATGA | GCTGAAGTTGCAGCACAGAC |
| *Xibmsp06* | HCO3 transporter family | CGGTGCTCATGTACACATTC | TGATAGCCTGCTGCATGAAG |
| *Xibmsp07* | Serine carboxypeptidase III precursor | GTCCCTTGCGTGGAACAAAT | AGCTAAAGCCAGTTCCAGTG |
| *Xibmsp08* | Serine carboxypeptidase | ACTTGACTCCAACCTCCAAC | TGGGGATACAGATGCTGTAG |
| *Xibmsp09* | Uridylate kinase | ATACGCCGAAGAGCTGTCAG | AGCGTAATGGCAGTCATGTC |
| *Xibmsp10* | Phosphatidylinositol 3-kinase | GCTGGAGCTTGACTCGTG | CAAAGAGAAACGAAATTTCCACA |
| *Xibmsp11* | Acetyl CoA carboxylase | CGTCAATGGCATATCTACAC | CCATACCAATGTCATTGAGC |
| *Xibmsp12* | Acyl CoA oxidase | TTTTGTTATCCACAGTCCAACTC | TGCCTTAGAAGCATCTGCAA |
| *Xibmsp13* | Potassium transporter | GGAAGTCGTAGCAGAAGTTG | CAAGGTCTCCATCAACTGGC |
| *Xibmsp14* | Serine-threonine protein kinase | TCTTCAGGGATGTTCCCTACT | GAGGAAGTTTATGATGGAAGGAAA |
| *Xibmsp15* | Zinc finger C-x8-Cx5-Cx3-H type | TGCTACGCCAATTTCTAATGC | CCACCATCGTCAAGTACTGC |
| *Xibmsp16* | Pitrilysin | GAGCTCCAGATGATGAACAC | CTTGCCATAGCACCAAATGG |
| *Xibmsp17* | MAP kinase | CATGGCACCACTAGACATAG | GAAACTGACTTCATGATGGAG |
| *Xibmsp18* | CBL interacting protein kinase | ATAGATAAAACAGGTGCAGTTTCAGA | ATGACCACAGATCAGCCTTG |
| *Xibmsp19* | 2-oxoglutarate dehydrogenase E1 component | GTGTTGGTTCCATCTCAGG | CTGCCTCATGGTTATGATGG |
| *Xibmsp20* | Succinyl-CoA ligase alpha subunit | GCTGAGCTTGACCTTGTTGTC | CCTGGCATGATTCCAATTTT |
| *Xibmsp21* | Hypothetical protein | GAACCTCATCCAACAATTCC | GCTGCTGATGTTGCTATTGC |
| *Xibmsp22* | *LHY* | CGAATCCTCTTGGTACCAAC | GATCGCTCTTCATGTGGTTC |
| *Xibmsp23* | Hypothetical protein | AAAGGACCAGTCACGTGAAG | ATAGCCTGGCCATTTCCTC |
| *Xibmsp24* | Ubiquitin conjugating enzyme | CATCATTGGCCCACACAAT | GAACAACTTAAGCTGGTAGATGC |
| *Xibmsp25* | Proteasome a-type and b-type | GTGAAAAAGGGTCCAAAGGG | GAAGCCCCAGTAAGTCTTC |
| SNP marker | Gene homology | Forward | Reverse |
| *Xibmsp26* | Catalase | GAGGTTCGTCAAGAGGTTCG | TCCTCGGCCTCAATAAGCTA |
| *Xibmsp27* | Alanine glyoxylate aminotransferase | CATTGCTCTTCATGGTGGAG | TGGAGCACTGAAGCCAGTAA |
| *Xibmsp28* | Glutaredoxin | CGGCCGAGGTACTAACAGTC | GAGAAGCTAGGGGCAACCTT |
| *Xibmsp29* | Delta-1-pyrroline-5-carboxylate synthetase | GATGCAAATTTGTGGGAACC | GCCGAGACTCGAAAACAATC |
| *Xibmsp30* | *FlO* | AGACAGACAGCACGCACAAC | GAGCTCGACGACATGATGG |
| *Xibmsp31* | *HD3* | ATCGATCTTGTGTGCAGTGG | GACCCGACATGAGGACATTC |
| *Xibmsp32* | Alcohol dehydrogenase 1 | CTGGTGACCATGTCCTTCCT | TTGGTGGTTTGGCAACATTA |
| *Xibmsp33* | ABA response protein | GAAGGAGAAGCACCACAAGC | CCGAGGATATCCAGATCGAA |
| *Xibmsp34* | *MADS*-box | GCTCGAAACACGAAACCCTA | CTGGCAGGTGACTTCTCCA |
| *Xibmsp35* | *MYC* | ACGAGATGTTCCTCGTCCTG | CCTCCTTGTTCGAGATGGTG |
| *Xibmsp36* | Opaque 2 | GCCAGTAAGGTCCATCTCCA | TAGCACGATCCACCTCATCA |
| *Xibmsp37* | *LEA* | ACAGCGTCTCCTTCAAGAGC | TCCGACTCCGATGTGGTACT |
| *Xibmsp38* | Vacuolar H^+^ ATPase subunit c | TACCTCTTCGATGGCTACGC | CTTGACACCACCAAAGACGA |
| *Xibmsp39* | RAB | ACCAACCAAGCCAACGAGTA | GGGTGGACTGAAATCGCTTA |
| *Xibmsp40* | Anion channel protein | ATGAGAAGCAGAAGGCCTCA | TCCAGATCCTTCCCACAGAG |
| *Xibmsp41* | Hydroxyproline rich-glycoprotein | TTTCTGCCGTCCATCTATCC | AAAGCGAAGGGCGTACTACA |
| *Xibmsp42* | Expressed protein | AGGCWGGAGAACTTGGAGCRCCAGTTG | GTGGYTTGCAGAAAGMCATATG |
| *Xibmsp43* | Actin depolymerising factor | AGTCSAARAGGMTGCACCGCTTCAT | TTGAACAGCGMAACACATGYCCATAG |
| *Xibmsp44* | Photolyase | CCAARTTTKAGAAACCTAAGGRTGACC | ACAAGCAACAGAGTGGCGTGCAAGATGATGCATCC |
| *Xibmsp45* | Expressed protein | GCAGCAGCAACCGCATCC | GCGGAKAGCTCRACRTCGCCGTGCTG |
| *Xibmsp46* | Plectin/s10 domain | CCATGATCATCYCCAAGAAGAACCGC | ACTCCTTKGACTTGAAGCTCTGCATGAGCT |
| *Xibmsp47* | Hypothetical protein | GGAGCTKGAGAAGAARTTCAGTGG | CTTSCCMACAATCTCAGCTGG |
| *Xibmsp48* | Thioredoxin peroxidase | GCTCTGAGAGGAYTRTTCATCATTGACAAGGAGGG | GGGTCRGGCTTCATYGACTTBTCCCCAGG |
| *Xibmsp49* | Atftsh2/8 | CTCCACAAACTCGGAAAGGA | ATTTGGAGAGCCTGAGGTGA |
| *Xibmsp50* | Fatty acid desaturase | CCCAAATGCTCTCTTTCCTG | CACCACCTCTTCTCCACCAT |
| *Xibmsp51* | Hypothetical protein | AAAGCTCTCAACGTCCCAGA | GCCCTCAACTCAGGCTTCTA |
| *Xibmsp52* | Expressed protein | ACGAGGTGCTCTTCATCTCC | GTCCGGATCGAATTCACATT |
| SNP marker | Gene homology | Forward | Reverse |
| *Xibmsp53* | PSI reaction center subunit III | GGCGATGTAGAGGAAGAGCA | TCCTCGCTCAAGAAGTACGC |
| *Xibmsp54* | Eucaryotic initiation factor 4A | GTACACCTGGGCGTGTCTTT | CTTGTCAGTGAGCCAGTCCA |
| *Xibmsp55* | *PHYC* | TTCCATCCCAACCATTCCTA | CCGGAACATCTCCTGGATAA |
| *Xibmsp56* | Elongation factor | CTGTCTTCCCAGCACACTACC | TGAGTCCTTTGGGTTCTCCA |
| *Xibmsp57* | Zn finger *WRKY* | CTTCAGCAGCCGATCAAAAT | ACCTCACTGGTTGTTTGCAC |
| *Xibmsp58* | Fe-S precursor protein | GCGAGCAGCATCYCSGCSGACCGCGTCCC | TGGCARGGGCAGAKGAACTTGTTCTCGGCG |
| *Xibmsp59* | Ycf68 | ACGAGGGGACAAGGTGGT | GGCGAGAGTTGTTTGGTTTC |
| *Xibmsp60* | Dipeptidyl peptidase IV | GTTTATGGYGGYCCCAGTGTCCAGCT | GCWAGGCCYTKCTTTATTARCCACTC |
| *Xibmsp61* | Peroxidase | ACCGGCTCTACAACTTCAGC | CCGCCACATCCATGTTTCTA |
| *Xibmsp62* | Actin | CAAGCAGCTGACGATGGTAC | GCTGAAAGAGCAGTATGCATG |
| *Xibmsp63* | AMP deaminase | ATCCTCAGCTCCATGTTTTC | ATTAGTCCATTGCTCAGGTG |
