# Supplementary material for: Integration of gene-based markers in a pearl millet genetic map for identification of candidate genes underlying drought tolerance quantitative trait loci
Source: BMC Plant Biol. 2012 Jan 17;12:9. doi: 10.1186/1471-2229-12-9 (PMC3287966; doi:10.1186/1471-2229-12-9)
Supplement: Additional file 2 — Table S2. Forward and Reverse pair of primer sequences used for CISP markers development. [file 1471-2229-12-9-S2.DOCX]

Table 2 Forward and Reverse pair of primer sequences used for CISP markers development

| Primer | Gene homology | Forward | Reverse |
| --- | --- | --- | --- |
| *Xibmcp01* | Heat Shock protein | AGGAGGTGAAGGTGGAGGTC | CATTGTTTCAACCCACATGC |
| *Xibmcp02* | Ribosomal protein L24 | GAGTAGAGCATCACATTTGAG | CATTGCAGGCCGTGAGAAAG |
| *Xibmcp03* | Transmembrane amino acid transporter | GGGTCCCTTTATAAACAAAAACA | CAAGGCACATTTCTCGTGAC |
| *Xibmcp04* | Transaldolase | CCCACTTCACAAGCTGCATA | CGGTTGTGTAGGCCGTTTAC |
| *Xibmcp05* | C2 domain | CTGAATGGAACGAGACCTTC | CTGCTGGAGTGAAGGTGAG |
| *Xibmcp06* | Adenosyl homocysteinase | CACCAAGCTTGCCCAAGTG | CATCATTGTCCTTGCTGAGG |
| *Xibmcp07* | Phosphate translocator | GCCGAATATGTATCCCTGCT | CAAGGCTAGGATGGAAGAGG |
| *Xibmcp08* | Phosphoglycerate kinase | TGTTGGCTTGAATGTTTTGG | GCCAGCTCAATTAGCTCCAG |
| *Xibmcp09* | Chlorophyll A/B binding protein | GCCATGCTCGCTGTGGTA | TACTTCTTCTTCTCGGGATCCTT |
| *Xibmcp10* | Delta-1-pyrroline-5-carboxylate synthetase | AGATAGCAAGCCTCGCAAAA | ATCAAGAGCACACCCAAAGG |
| *Xibmcp11* | Protein phosphatase 1 regulatory subunit SDS22 | CAAGAGCTATGGCTGGGAAG | AGACCTTCCATCTTTTGAATGC |
| *Xibmcp12* | Expressed protein | TGTTAGCCCTTGTTCCTCCA | AGCAACACCAGCTGACCTTT |
